# Immediate, Early, and Conventional Implant Placement in a Patient with History of Periodontitis

**DOI:** 10.1155/2015/217895

**Published:** 2015-04-08

**Authors:** Alessandro Lanza, Fabio Scognamiglio, Felice Femiano, Michele Lanza

**Affiliations:** ^1^Multidisciplinary Department of Medical, Surgical and Dental Sciences, Second University of Naples, Via Luigi De Crecchio 7, 80138 Naples, Italy; ^2^University of Naples Federico II, 80138 Naples, Italy

## Abstract

The aim of this paper is to describe a case of implant-prosthetic rehabilitation in a patient with periodontitis, focusing on the different timing of implant placement. After initial periodontal treatment, teeth with advanced mobility degree and severe bone resorption were extracted. At different healing time oral implants were placed in a prosthetic-guided position. After osseointegration period the implants were loaded and the results at one year of follow-up are presented.

## 1. Introduction

The implant treatment represents a valid therapeutic option to replace lost teeth due to various causes with high success and survival rates at medium-long term [1]. However, according to the Seventh Workshop on Periodontology [2], the risk of complications and peri-implant infection exists and increases in subjects with periodontitis, with poor oral hygiene and smokers [3]. Therefore, the control of these risk factors and periodontal therapy are key elements to achieve stable results over time [4]. The scientific literature and clinical experience show that, before any rehabilitative intervention, periodontal treatment must be carried out in all cases where it is necessary and leads to a higher probability of implant success [5–7]. A recent prospective longitudinal study shows that the risk of infection increased after ten years in case of treated periodontitis, especially if aggressive [8]. However, the periodontal therapy and motivational reinforcement to oral hygiene represent valid elements to reduce the risk of peri-implant infection and the need for surgery in case of peri-implantitis. After long periods of observation, the risk of peri-implantitis increases in patient with history of periodontal disease, especially in case of smoker patient. Hardt et al. reported a 5-year implant success rate of 97% in periodontal healthy patients and 92% in periodontal patients [9]. He also reported that 64% of patients with a history of periodontal disease had a mean peri-implant bone resorption greater than 2 mm from the delivery of the prosthesis, compared to 24% in periodontal healthy patients. Mengel et al., moreover, in a prospective longitudinal clinical study compared the results obtained in 15 patients treated for generalized aggressive periodontitis, in 12 patients treated for generalized chronic periodontitis, and finally in 12 periodontal healthy patients considered [10]. In periodontal healthy patients and in patients with chronic periodontitis implant success rates were equal to 100% while in patients with aggressive periodontitis a success rate of 95.7% in the maxilla and 100% in the mandible was achieved. In the literature also other factors that can influence the outcome of implant treatment have been analyzed. Among these, the timing of load result is not able to influence the therapeutic outcomes (implant failure: RR 1.65; 95% CI 0.68 to 3.98; 10 trials) [11]. Even for the timing of implant placement there is insufficient evidence to determine the possible advantages or disadvantages of immediate, immediate-delayed, or delayed implants; therefore the preliminary conclusions of the literature are based on few underpowered trials often judged to be at high risk of bias. In the Third International Team for Implantology (ITI) Consensus Conference, three basic protocols for implant placement were defined according to the time between tooth extraction and implant installation [12]. In the type-1 protocol (immediate implant installation), implants are placed in fresh extraction sockets, with the aim to engage the remaining socket walls with the implant. In the type-2 protocol (early implant placement), implants are placed approximately 4–8 weeks after tooth extraction. The main objective of this protocol is to ensure the lack of pathology when placing the implant and, at the same time, to optimize the availability of soft tissue for primary healing and probable lateral bone augmentation. In the type-3 protocol (early-delayed/conventional implant placement) the implants are placed once most of the dimensional changes in the alveolar ridge have occurred (12–16 weeks). Chen et al. have analyzed the survival rates and clinical outcomes of immediate and early postextraction implants with implants placed in healed sites and concluded that there are no statistically significant differences in terms of implant survival in the short term (12 months of follow-up) [13]. Even the Quirynen's group in a recent review on this topic leads to the same conclusion by asserting that in the absence of long-term data, the clinical outcomes of postextraction implants and implants placed in healed sites are practically the same [14].

The fixture placement in relation to the dental extraction should be based on an adequate understanding of the structural changes that occur in the alveolar process after tooth loss [15]. During the healing, alveolar bone walls are partly absorbed, the center of the socket is filled with porous bone, and the overall volume of the site is greatly reduced [16]. The buccolingual resorption, however, prevails and occurs in the first 3 months from the extraction [17, 18].

Therefore, the clinician must know and predict the changes of postextraction ridge to plan the timing of implant placement and the management of the site in the best way possible in order to get predictable results. In light of these premises, we describe a clinical case that came to our attention to be rehabilitated by dental implants due to the loss of teeth in the maxillary and mandibular posterior region for severe periodontitis. The aim of this work is to illustrate the importance of periodontal disease infection control together with prudent management of the timing of implant placement in different sites in order to best solve the problem of the patient.

## 2. Clinical Case Report

**Figure 1:**
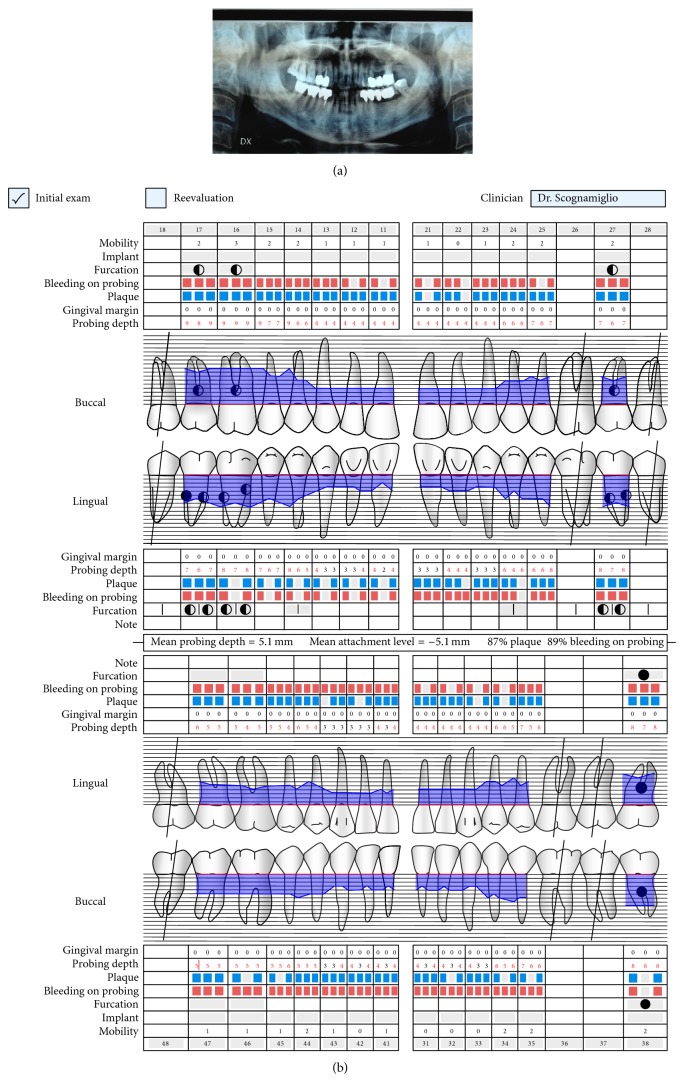
(a) Initial OPT radiographic situation; (b) initial periodontal charting.

**Figure 2:**
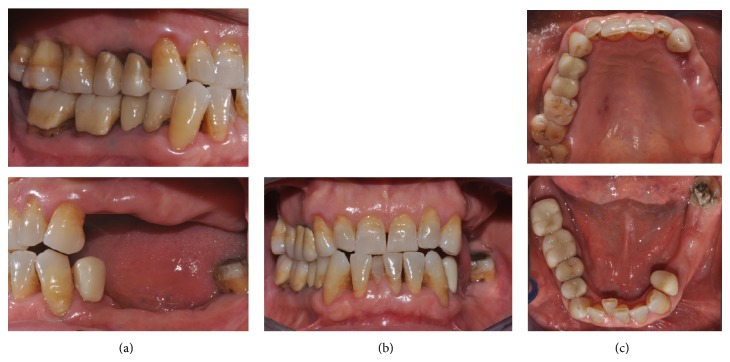
Clinical condition after periodontal treatment: (a) lateral view, (b) frontal view, and (c) occlusal view.

**Figure 3:**
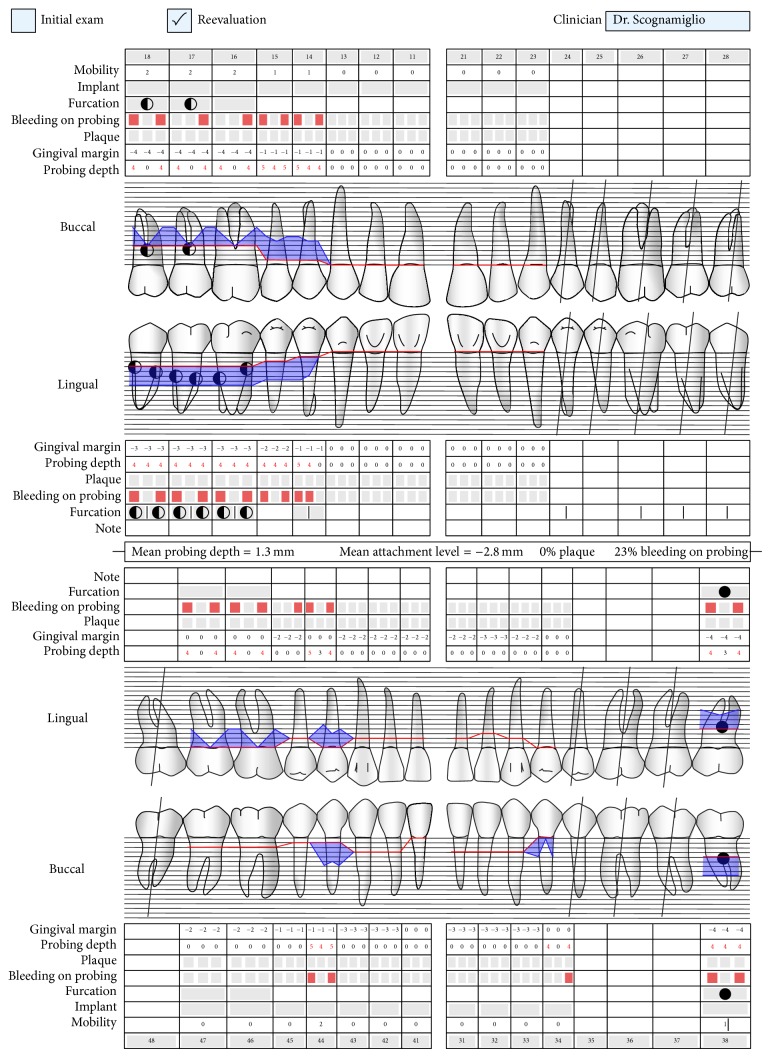
Periodontal condition after nonsurgical periodontal treatment.

**Figure 4:**
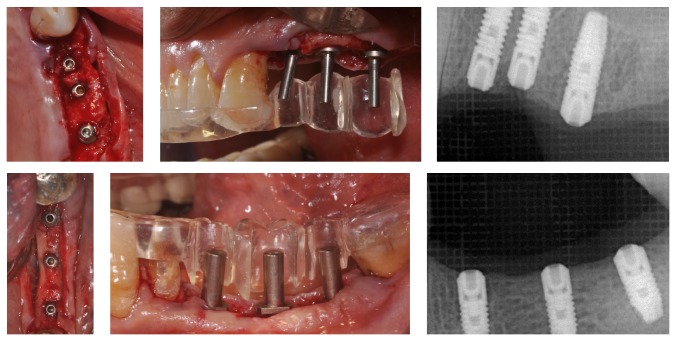
Type 3 prosthetically guided implant positioning, surgical and radiographic view.

**Figure 5:**
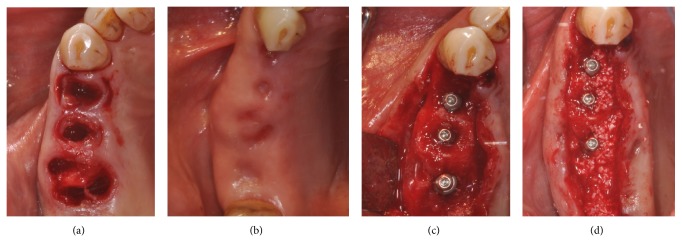
Type 2 implant positioning with simultaneous GBR technique: (a) extraction time point, (b) 4-week clinical soft tissue healing condition, (c) implant insertion, and (d) simultaneous GBR to optimize bone volume.

**Figure 6:**
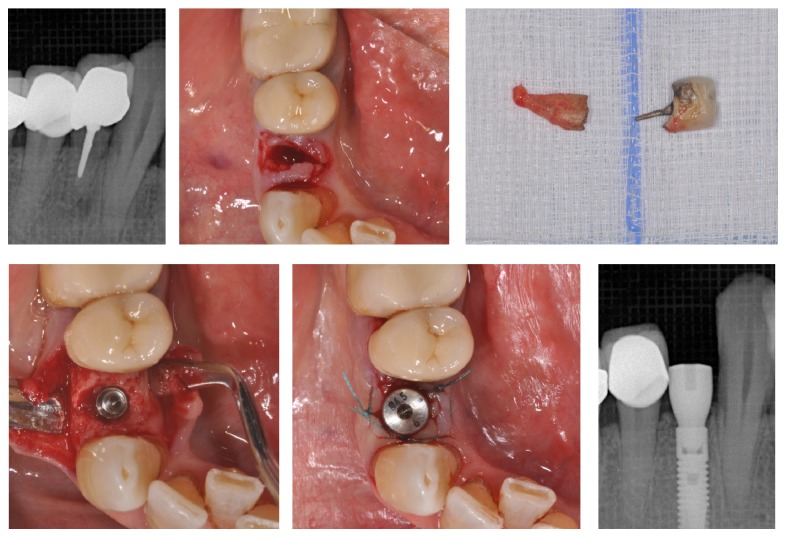
Type 1 implant positioning with transmucosal healing, surgical and radiographic view.

**Figure 7:**
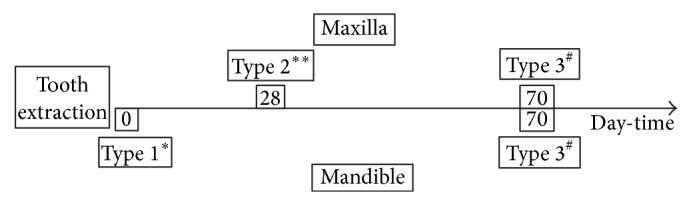


**Figure 8:**
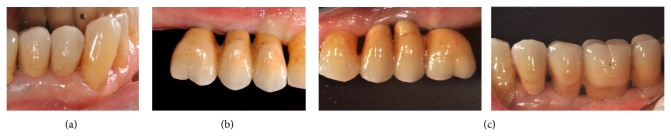
Clinical situation at the delivery of prosthetic rehabilitation: (a) type 1 implant positioning; (b) type 2 implant positioning; (c) type 3 implant positioning.

A 63-year-old patient suffering from advanced chronic periodontitis came under our observation to resolve his dental problems with a specific request to be rehabilitated with fixed prostheses. The patient was in good health general state. Intraoral examination showed tartar deposits and various mobility degrees of the upper and lower teeth in the masticatory region. In addition, there were no adequate fixed restorations on natural teeth numbers 4-5-28-29-30-31. In Figure 1 the initial periodontal charting and radiographic status of the patient are shown. The panoramic radiograph presented by the patient before avulsion of the left side elements indicated widespread bone resorption. After taking alginate impressions, models were developed with diagnostic wax-up of the case. A TC Dental Scan to evaluate the available bone volume for the prosthetic rehabilitation of periodontally compromised sites has been prescribed. After careful oral examination and periodontal charting, nonsurgical periodontal therapy was carried out and reevaluation at 4 weeks was done. At this time point, the patient had severe periodontal bone loss in the posterior right regions. The images demonstrate the clinical healing at 10 weeks (Figure 2) after the extractions of left posterior teeth. Periodontal charting at reevaluation is shown in Figure 3. On tooth number 21 we proceeded to remove the incongruous fixed prosthetic crown and we cemented a provisional resin restoration. The patient throughout the course of therapy was maintained periodontally stable and at each control was motivated to oral hygiene; this is in accordance with the guidelines set out recently in the seventh European Workshop on Periodontology [2]. After the reduction of local irritative factors implant treatment was undertaken (Figure 4). Surgery was performed under local anesthesia using 4% articaine solution combined with a vasoconstrictor (Ubistesin forte, 3M ESPE). The incision was extended from the edentulous distal crest to the sulcus of the mesial tooth, the canine. A full thickness flap was carefully elevated. It was decided to proceed with the placement of implants in sites numbers 12-13-14 (OsseoSpeed Astra Tech AB, Mölndal, Sweden) and numbers 21-20-19 with a type 3 implant placement (large bone healing and full maturity of soft tissue/T, 10 weeks). Before surgery, the patient has performed for 2 minutes rinse with chlorhexidine 0.20% (Curasept, Curadent Healthcare SPA, Saronno VA, Italy). The prosthetic-guided implant positioning was obtained with the aid of a surgical template developed by the initial diagnostic wax-up (Figure 3). Sites numbers 12-13-14 were prepared to accommodate 2 implants, respectively, 3.5 × 11 mm and 1 implant 4.0 × 11 mm. In the mandible 3 implants were inserted: 3.5 × 9 mm (number 21), 3.5 × 11 mm (number 20), and 4.0 × 9 mm (number 19), respectively. The wound was closed with E-PTFE mattress and simple sutures (expanded polytetrafluorethylene, Gore-Tex, W. L. Gore Associates Inc., Flagstaff, AZ 86004). After surgery, the patient received antibiotic prophylaxis with amoxicillin 2 g/day and rinsed 0.2% chlorhexidine digluconate 3 times/day for 4 weeks. The patient was shown how to perform a roll-stroke brushing technique and was motivated to control oral hygiene. The patient did not report specific symptoms and showed no adverse clinical signs. During implant osseointegration implant, the patient did not apply on the edentulous ridges any provisional prosthetic rehabilitation in order to avoid trauma and wound dehiscence in the early stages of healing. After 5 months of healing, we proceeded to expose the implants with a small incision using a miniblade. The cover screw was replaced with a healing abutment. We proceeded at 7 days to take the polyether impression (Impregum Penta 3M ESPE). Clinical examination at the delivery of the prosthesis revealed clinically healthy peri-implant soft tissue and no signs of complication. We proceeded to cement with temporary device (Temp Bond, Kerr, Italy) carefully controlling cement excess to avoid inflammation of peri-implant tissues [19]. After 4 weeks, we proceeded to rehabilitate the posterior right regions. For the maxillary arch implant placement was made at six weeks from the extractions (type 2 placement). Soft tissue healing made easier the surgery but the partial bone healing has forced us to a recountourning of the crest with a xenograft (Bio-Oss, Geistlich, Wolhusen, Switzerland) at the gap created between the implant surface and the residual alveolar extraction cavity (Figure 5). In fact, literature demonstrates a spontaneous healing for 4-wall defects with bone gap of 1.5–2 mm [20]. In cases of osseous irregularity or big defects is necessary to use a bone graft to counteract the peri-implant tissue remodeling. The width of the ridge was well maintained, showing postoperative ridge width between 5 and 6 mm at the most coronal portion of the alveolar bone. Two 3.5/13 mm implants were inserted in the number 5-4 sites and 1 implant 4.0/9 mm in site number 3. One month after the surgery we removed incongruous fixed prosthesis on numbers 31-30-29, grinded stumps, and delivered provisional bridges number 31 to number 29. After two weeks, number 28 was extracted because fractured and an immediate implant was placed in fresh socket (type 1) (Figure 6). In Table 1 all implant dimensions and a brief surgical and prosthetic schematic overview are shown. Here we have opted for a transmucosal healing for an easier management of the flaps and tissue closure. Literature does not demonstrate statistically significant differences in terms of survival and success rates for implants inserted with two-stage or one-stage technique and transmucosal healing [21, 22]. The osseointegration period was, respectively, 6 months for the maxilla and 4 months for the mandibula. All postextraction implants were inserted by placing implant shoulder 1 mm under the crestal bone level providing the physiological ridge alterations after extraction, and were anchored to the lingual/palatal cortical plate of the post-extraction sites [15–18]. In Figure 7 is shown a schematic overview of timing of implant placement relative to tooth extraction. Clinical control at the delivery of the prothesis shows the appearance of a great aesthetic result and good quality of peri-implant soft tissues (Figure 8).

## 3. Discussion

**Figure 9:**
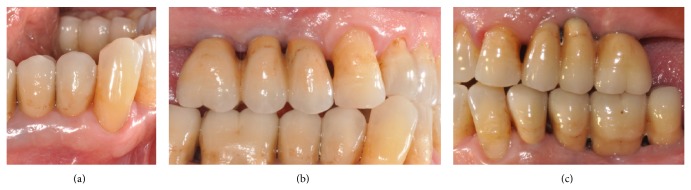
Clinical situation after 1 year from loading time; (a) type 1 implant positioning; (b) type 2 implant positioning; (c) type 3 implant positioning.

**Figure 10:**
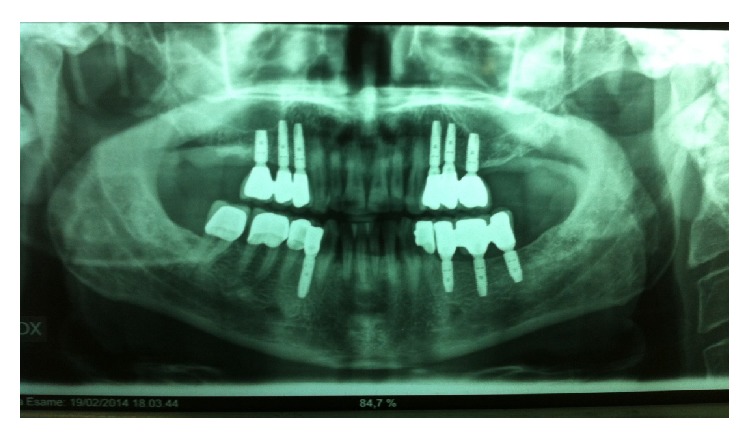
OPT radiographic situation 1 year after loading.

The use of implants in patients with teeth lost due to periodontal disease is now established practice [23, 24]. However the risk of failure increases in patients with periodontitis, smoking, and poor oral hygiene [8]. In agreement with the Sixth European Workshop on Periodontology implants were placed to support fixed prosthesis, after successful completion of initial periodontal therapy (full-mouth plaque score (FMPS) <25%, full-mouth bleeding score (FMBS) <25%) and the patient receives proper periodontal maintenance care [4]. The careful management of the implant insertion timing in this implant-supported rehabilitation type allowed to minimize the risk of complications and to achieve good results as confirmed by the scientific evidences [3]. However, the significant bone reduction resulting from the tooth extraction and the vertical defects typical of this patient affected by periodontal disease led to a not aesthetically perfect result. This outcome was expected and we properly informed the patient before starting the treatment, because we could not get a complete coverage of the dental papilla triangular space for increasing the distance between the interproximal bone crest and the contact point [25, 26]. However, the correct three-dimensional implant placement and the management of the peri-implant tissue have allowed us to obtain a satisfactory result. The integrity of the buccal cortical bone in the case of postextraction implants is a fundamental prerequisite because the contact between the implant and the thin buccal bone exposes us to an inevitable consequence represented by a more pronounced bone resorption and thus more rapid exposure of the implant surface. Experimental and clinical studies have shown that the placement of postextraction implants does not alter the pattern of bone resorption after dental extraction [27, 28]. After tooth loss, there is a reduction of bone quantity and the immediate implant insertion does not alter this physiological phenomenon [29]. However, the use of small diameter fixtures and their correct positioning (more lingual) together with simultaneous or delayed guided bone regeneration techniques may help the clinician to maintain and preserve the peri-implant tissue volume improving clinical and aesthetic outcomes even in case of postextraction implants [15, 30]. Another difficulty linked to the postextraction implant placement is to obtain the primary stability in a prosthetically guided position. In type 1 and type 2 implant timing placement, the fixtures are anchored apically to alveolar socket in native bone. However in a controlled clinical trial, Siegenthaler, noted that with a protocol of immediate implant placement did not always achieve the primary stability of the implants [31]. So, in the presence of large sockets that preclude the involvement of apical native bone, it may be preferable to defer implant placement. In this specific case, the implant insertion along the axis of the future prosthetic reconstruction expected has allowed us to obtain a good primary stability in the sites numbers 3-4-5 and number 28. There were no complications and all fixtures were loaded after a conventional healing period of 3–6 months. Respect for the hard and soft tissues, the maintenance of a good oral hygiene together with the use of premium materials has allowed us to achieve good clinical and radiographic results observed 12 months after delivery of the prosthesis (Figure 9). The radiographic examination 1 year after the delivery of the prosthesis demonstrates the absence of peri-implant bone loss (Figure 10).

## 4. Conclusion

When teeth have to be replaced by oral implants, there are various factors conditioning the timing of implant placement after dental extraction, among these, the three-dimensional position of the tooth in the oral cavity, the hard and soft tissue contour of the site, and the adaptive changes of the alveolar ridge after tooth extraction that may affect the outcome of the therapy. The decision on the planning for implant placement should be based on a full understanding of the structural changes that occur in the alveolar process after tooth extraction, with and without implant placement as pointed out by the scientific evidence on this topic. Another parameter to be considered is the well knowledge of patient oral and periodontal conditions (Table 2). Periodontal disease should be treated completely before implant treatment. In addition, the patient should be monitored over time in order to reduce inflammatory indexes that may increase the failure risk and biological complications of our implant-supported restorations. Regarding implant placement timing, it is hard to provide clear evidence because of the paucity of studies that compare the various methods of implant insertion and for the presence in the literature of clinical trials with high risk of bias. However, within the limit of this case report, timing of implant placement does not represent a parameter that can affect the short-term treatment outcomes if you meet certain principles.

## Figures and Tables

**Table 1:** Surgical and prosthodontic schematic overview.

Timing	Days after teeth extraction	Site	Fixture height/diameter	GBR+/−	Healing model	Loading time
Type 1	0	Mandible	13 mm/3.5	−	Transmucosal	3 months

Type 2	28	Maxilla	13 mm/3.5 13 mm/3.5 9 mm/4.0	+	Submerged	4 months

Type 3	70	Mandible	9 mm/3.5 11 mm/3.5 9 mm/4.0	−	Submerged	3 months 3 months
		Maxilla	11 mm/3.5 11 mm/3.5 11 mm/4.0			

See Figure 7.

**(a):** 

Presurgical periodontal conditions				
Suppuration	FMPS	FMBS	Tooth mobility	Pocket ≥ 5 mm
None	<25%	<25%	0/1^∗^	Numbers 5-4-3-2
			2/3^∗∗^	

^∗^Tooth mobility in aesthetic region and on numbers 29-30-31.

^∗∗^Tooth mobility on numbers 5-4-3-2-28.

**(b):** 

1 year follow-up				
Suppuration	FMPS	FMBS	Tooth mobility	Pocket ≥ 5 mm
None	<25%	<25%	0/1^∗^	None

^∗^Tooth mobility in all sites.
